# Biomarkers predicting the efficacy of immune checkpoint inhibitors in hepatocellular carcinoma

**DOI:** 10.3389/fimmu.2023.1326097

**Published:** 2023-12-22

**Authors:** Ran Qin, Tianqiang Jin, Feng Xu

**Affiliations:** Department of General Surgery, Shengjing Hospital of China Medical University, Shenyang, China

**Keywords:** immunotherapy, hepatocellular carcinoma, biomarker, PD-L1, HCC, immune checkpoint inhibitor

## Abstract

In recent years, immune checkpoint inhibitors (ICIs) have emerged as a transformative approach in treating advanced hepatocellular carcinoma (HCC). Despite their success, challenges persist, including concerns about their effectiveness, treatment costs, frequent occurrence of treatment-related adverse events, and tumor hyperprogression. Therefore, it is imperative to identify indicators capable of predicting the efficacy of ICIs treatment, enabling optimal patient selection to maximize clinical benefits while minimizing unnecessary toxic side effects and economic losses. This review paper categorizes prognostic biomarkers of ICIs treatment into the following categories: biochemical and cytological indicators, tumor-related markers, imaging and personal features, etiology, gut microbiome, and immune-related adverse events (irAEs). By organizing these indicators systematically, we aim to guide biomarker exploration and inform clinical treatment decisions.

## 1 Introduction

Hepatocellular carcinoma (HCC) is one of the most common malignant tumors, with the fifth highest incidence and third highest mortality rate of all malignancies globally (1–3). Treatment options, including hepatectomy, liver transplantation, ablative therapies, and various therapies, offer hope for early-stage patients; however, late-stage diagnoses limit surgical options. Consequently, systemic therapies, often involving conventional chemotherapy, remain the primary approach, despite their suboptimal effectiveness due to HCC’s drug-resistant nature (4). Therefore, the urgent need for novel drugs and strategies arises.

Over the last decade, multitargeted tyrosine kinase inhibitors (TKIs) like sorafenib and lenvatinib have yielded a certain degree of improvement in the survival of HCC patients, with median overall survival (OS) ranging from 11 to 14 months (5–7). In recent years, immune checkpoint inhibitors (ICIs), including anti-programmed cell death -1/programmed cell death ligand-1 (PD-1/PD-L1) and anti-cytotoxic T lymphocyte-associated antigen-4 (CTLA-4) agents, have revolutionized the management of cancer (8–10). Although initial studies on nivolumab and pembrolizumab showed promise, later phase III clinical trials failed to meet their primary endpoints (11, 12). However, in the Phase III clinical trial IMbrave150, the combination of anti-PD-L1 and anti-vascular endothelial growth factor receptor (VEGFR) agents, atezolizumab plus bevacizumab (Atez/Bev), demonstrated significant OS improvement compared to sorafenib, becoming a recommended first-line treatment for advanced HCC (13). Additionally, recent HIMALAYA research revealed that the tremelimumab and durvalumab combination outperformed the sorafenib treatment, also gaining recognition as a first-line option for advanced HCC (14).

Despite these remarkable advancements, not all patients respond favorably to treatment. Even in patients receiving combination immunotherapy, 20% of patients are refractory to Atez/Bev, with only 20%-30% showing radiological responses. In addition, a considerable number of patients experience grade 3–4 immune-related adverse events (15, 16). Therefore, understanding the determinants of treatment response and adverse effects is crucial. As HCC systemic treatment options continue to increase, the ability to predict treatment response and survival benefits has become an exigent necessity.

**Figure 1:**
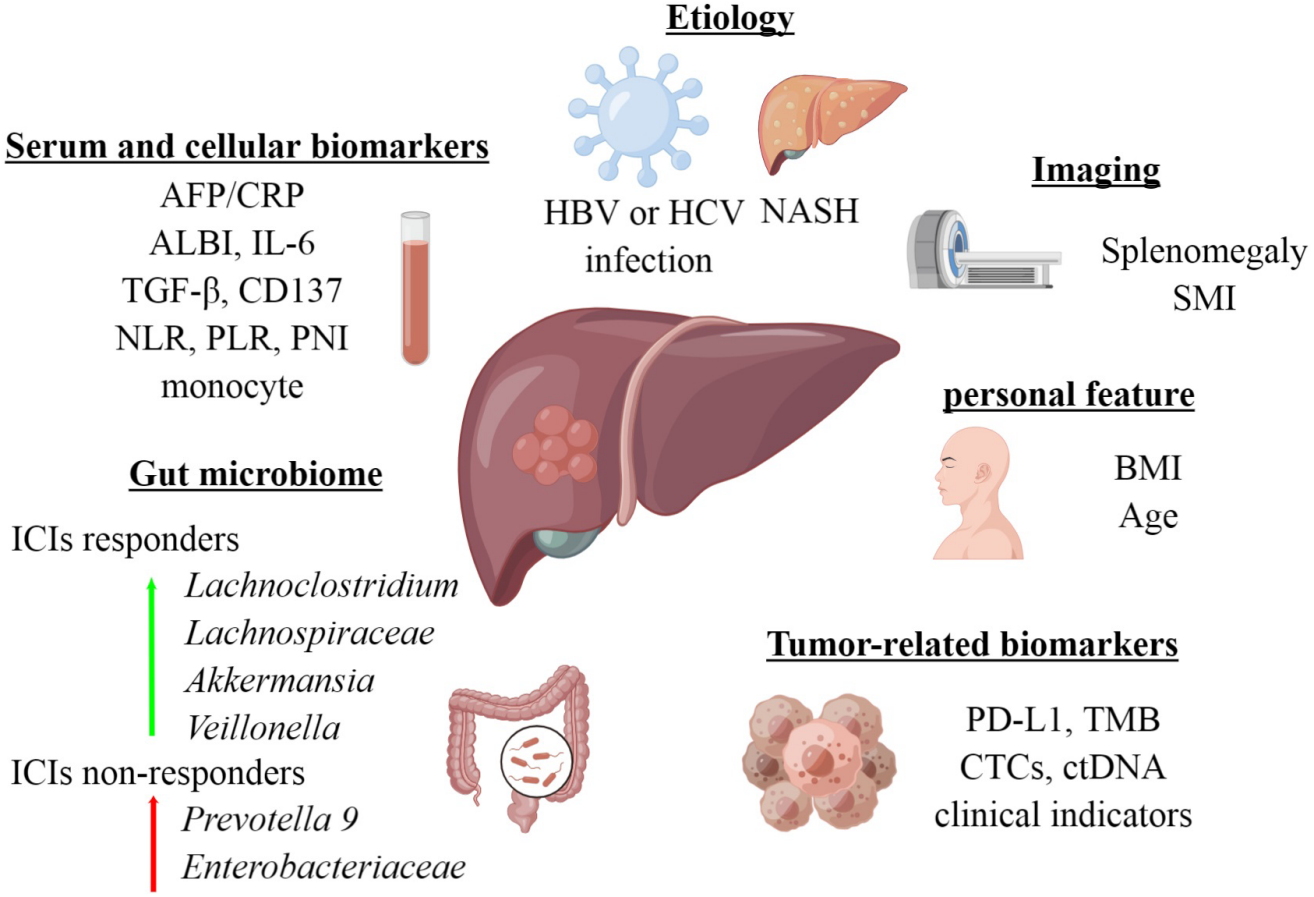
Biomarkers of response to immune checkpoint inhibitors in HCC. Created by Figdraw.

In this review, we summarize the recent clinical data on the efficacy prediction of ICIs for HCC, to provide valuable guidance for optimal treatment selection (**Figure 1**).

## 2 Serum and cellular biomarkers

### 2.1 Predictive biomarkers before treatment

#### 2.1.1 AFP and CRAFITY score

Alpha-fetoprotein (AFP) is a widely used biomarker for the diagnosis and monitoring of efficacy in HCC (17, 18). Recent studies have revealed its association with the effectiveness of ICIs in HCC. In a retrospective multicenter study involving 99 patients receiving nivolumab or pembrolizumab, those with AF*P<*400 µg/L before treatment demonstrated a better response and prolonged survival [median progression-free survival (PFS): 5.4 vs. 2.6 months, *P* < 0.05; median overall survival (OS): 21.8 vs. 8.7 months, *P* < 0.0001] (19). Another study corroborated these findings (20). A meta-analysis of 44 retrospective studies further highlighted that elevated AFP levels correlated with shorter OS and PFS, as well as a lower disease control rate (DCR) compared to lower AFP levels (21). Additionally, the CRAFITY score, combining baseline AFP and C-reactive protein (CRP), demonstrated efficacy in predicting ICIs response. Within this scoring system, patients with AFP≥100 ng/ml and CRP≥1 mg/dl received 2 points, those meeting one criterion scored 1 point, and patients not meeting the criteria scored 0 points. The median OS and DCR for patients with 2 points were significantly better than those with 1 point or 0 points (median OS: 27.6 months, 11.3 months, and 6.4 months, respectively; DCR: 80%, 64%, and 39%, respectively, P < 0.001) (22). The predictive capability of the CRAFITY score was further validated in multicenter studies conducted in different countries. Yang et al. suggested that the CRAFITY score could predict the survival prognosis of HCC patients treated with ICIs combined with TKIs (23). Hatanaka et al. also confirmed its predictive value in Atez/Bev treatment for DCR, OS, and immune-related adverse events (irAEs) (24). In conclusion, the CRAFITY score serves as a simple and effective predictive indicator, readily obtainable and applicable. However, it is important to consider CRP’s susceptibility to injury or infection, given its nature as an acute-phase protein. Further prospective studies are needed to verify its clinical value.

#### 2.1.2 ALBI score

HCC mainly affects patients with advanced liver fibrosis or cirrhosis, indicating that liver function impairment significantly impacts prognosis (25). The albumin-bilirubin (ALBI) score, a simplified method for assessing liver function in HCC patients, categorizes patients into grades 1-3, with grade 1 representing the least liver damage (26). A prospective study demonstrated that the pre-treatment ALBI score can independently predict OS and serve as a stratifying biomarker for ICIs treatment (27). Another study investigated HCC patients who experienced tumor progression after prior ICIs treatment and subsequently received ipilimumab plus nivolumab or pembrolizumab, finding that all responders had a baseline ALBI grade of 1 or 2, indicating relatively preserved liver function. The study also observed a negative correlation between the ALBI score and OS (28). In a retrospective study, the baseline ALBI score and age were identified as independent prognostic factors influencing PFS and OS in HCC patients receiving ICIs combined with radiotherapy. The low-risk group (ALBI grade 1 and age≥53 years) exhibited significantly superior survival outcomes compared to the high-risk group (median OS: not reached vs. 10.1 months, *P<*0.005; median PFS: 15.3 months vs. 2.7 months, *P<*0.005) (29). A systematic review involving 4483 patients also revealed that the ALBI score could predict prognosis (30). Collectively, these studies underscore the significant importance of the ALBI score in selecting appropriate populations for ICIs treatment.

#### 2.1.3 Cytokines

Cytokines, such as interleukin 6 (IL-6), interferon alpha (IFNα), transforming growth factor-β (TGF-β), and others, have been investigated as potential biomarkers for predicting ICIs treatment response in various cancers, including HCC (31, 32).

IL-6, an inflammatory factor associated with HCC development and progression, has been studied in relation to ICIs treatment (33). High baseline serum IL-6 levels were found to be an independent risk factor for disease progression in patients receiving Atez/Bev, resulting in significantly shorter PFS and OS compared to those with low levels. Additionally, the study also demonstrated that patients with high baseline IFN-α levels had shorter OS compared to those with low levels (34). Another multicenter prospective research of 165 patients also showed that high baseline IL-6 levels were associated with a reduced response rate and worse PFS (HR= 2.93, *P* = 0.003) and OS (HR= 3.02, *P* = 0.021) (35). However, it is worth noting that IL-6 levels may be influenced by other inflammatory conditions, reducing its specificity as a predictive biomarker.

TGF-β, central in inflammation, fibrogenesis, and immunomodulation in the HCC microenvironment (36), has shown potential as a predictive biomarker for ICIs efficacy. In a study of 28 unresectable HCC patients treated with pembrolizumab, among several plasma biomarkers, only baseline TGF-beta cytokine levels in peripheral blood were significantly higher in non-responders compared to responders. Patients with baseline TGF-β levels higher than 200pg/mL had poor treatment response and shorter PFS and OS (37).

CD137, a cell surface marker expressed on activated T cells, has also shown promise as a biomarker for ICIs response. Patients with higher serum CD137 concentrations demonstrated significantly better clinical benefit and longer PFS (median PFS, 14.2 months vs. 4.1 months, *P*=0.001) (38).

While these findings highlight the potential of cytokines as biomarkers for predicting ICIs response in HCC, additional research and larger prospective studies are needed to confirm their utility, establish their clinical significance, and determine their specificity in predicting treatment outcomes.

#### 2.1.4 Peripheral blood cell

Peripheral blood components play a crucial role in various diseases and have been utilized to assess mortality risk in patients across multiple conditions, including HCC (39–42).

In a prospective study of 34 patients treated with Atez/Bev, the baseline percentage of peripheral granulocytes and their PD-1 and PD-L1 expression emerged as predictors of patients’ response and prognosis, with lower PD-1+ granulocyte percentage associated with a better response and longer time to progression (43).

The neutrophil to lymphocytes ratio (NLR), an inflammatory marker, has gained attention in HCC (44). Elevated NLR is believed to contribute to HCC recurrence by creating a protumorigenic microenvironment through both relative neutrophilia and lymphocytopenia (45). Recent studies have found that NLR is related to the prognosis of ICIs treatment (45, 46). Notably, a study by Dharamapuri et al. revealed that in HCC patients treated with nivolumab, having a pre-treatment NLR<5 (23 months vs. 10 months, *P=*0.004) and a post-treatment NLR<5 (35 months vs. 9 months, *P<*0.001) were significantly associated with improved OS (47). Another study confirmed that patients with NLR≥5 had poorer objective response rate (ORR), OS, and PFS (48). Furthermore, NLR was identified as a facilitating factor for pembrolizumab response in a single-arm prospective phase 2 clinical trial (49). Moreover, Tada et al. found that NLR before treatment (cut-off value of 3) was an independent predictor of response in HCC patients treated with Atez/Bev and was negatively correlated with OS (50). A meta-analysis also demonstrated that patients with high NLR levels had significantly poorer OS and PFS, lower ORR and DCR, and higher hyperprogressive disease (21). Moreover, the Advanced Lung Cancer Inflammation Index (ALI), calculated as body mass index (BMI) * Albumin/NLR, has the potential as a predictive biomarker. A retrospective study involving 98 patients found that high ALI was an independent prognostic factor for OS, with a hazard ratio of 0.411 (51).

The prognostic nutritional index (PNI), incorporating peripheral blood lymphocyte count and serum albumin levels, reflects the nutritional and immune status of the body. An analysis of 442 patients receiving anti-PD-1 monoclonal antibody treatment demonstrated that baseline NLR, PLR, and PNI all exhibited significant predictive ability for OS (HRs: 1.714, 1.691, 2.153, respectively; *P <*0.001). However, in multivariate analysis, only PNI was an independent predictor of OS: higher baseline PNI was associated with a poorer prognosis (HR = 1.770, *P <*0.001) (52). Changes in PNI after treatment can also serve as a predictor of ICIs treatment efficacy. In HCC patients treated with anti-PD-1 monoclonal antibodies, the PNI decreased in the progressive disease (PD) group after treatment compared to the non-PD group(*P* = 0.023), and an increased PNI was associated with longer OS (*P* = 0.014) (53).

The platelet lymphocyte rate (PLR) also has potential predictive value for the prognosis of HCC patients receiving ICIs treatment. A study has shown that patients with a baseline PLR≥300 have shorter median OS (6.4 months vs. 16.5 months) and PFS (1.8 months vs. 3.7 months). Moreover, PLR remains an independent prognostic factor for both OS and PFS (48). A high PLR after treatment is associated with a negative impact on survival prognosis (HR=1.002, *P<*0.001) (47).

The baseline monocyte-to-lymphocyte ratio (MLR) has been associated with ICIs effectiveness. In patients treated with anti-PD-1 monoclonal antibodies, the low MLR group exhibited a longer tumor progression time, with a median metastasis time of 33 weeks compared to 18 weeks in the high MLR group when disease progresses (54).

The circulating immune index (CII), calculated as the ratio of white blood cell counts to lymphocyte proportion, has also shown promise in HCC prognosis. A retrospective study involving 129 patients treated with ICIs and lenvatinib demonstrated that patients with CII ≤ 43.1 reported prolonged OS compared to those with CII > 43.1 (24.7 vs. 15.1 months, *P* = 0.019). CII was identified as an independent prognostic factor for OS. Moreover, patients with low CII levels demonstrated improved ORR and DCR (55).

The systemic inflammation response index (SIRI), calculated based on the absolute values of neutrophils, monocytes, and lymphocytes, has revealed evidence as an independent prognostic factor for HCC patients receiving sorafenib and/or ICIs treatment in a retrospective real-world study. High SIRI levels were associated with a poor response to ICIs, and SIRI is negatively correlated with peripheral CD3+, CD4+, and CD8+ T cell counts (56), indicating its potential link to immune cell activity.

These findings indicate that peripheral blood cell indicators hold potential as biomarkers for predicting ICIs treatment response and prognosis in HCC patients. Further research is necessary to validate their utility and explore the clinical significance in HCC management.

#### 2.1.5 Other serum proteins

In addition to AFP, which has been widely studied, several novel serum proteins have shown potential as biomarkers in HCC patients treated with ICIs.

Baseline low serum CXCL9 (<333 pg/mL) levels may predict early PD in patients with unresectable HCC treated with Atez/Bev (57). Patients with lower serum CXCL9 (<333 pg/mL) experienced early PD in 35.3% of cases(12/34) with Atez/Bev, resulting in significantly shorter PFS relative to those without early PD(median PFS, 126 days vs. 227 days; HR: 2.41, *P*=0.0084).

The Schlafen (SLFN) family members play an important role in oncology and immunity (58). A study by Zhou et al. observed that SLFN11 was up-regulated in ICIs-responsive HCC patients, and knockdown of SLFN11in HCC cells led to increased macrophage migration and M2-like polarization, indicating increased immunosuppressive macrophage infiltration. Furthermore, ICIs were more effective in HCC patients with high serum SLFN11 levels, suggesting SLFN11 as a potential predictive biomarker for ICI response in HCC patients (59).

In a retrospective study involving HCC patients treated with lenvatinib plus ICIs, two independent risk factors for OS were identified: Vitamin K absence or antagonist-II (PIVKA-II) and metastasis. To enhance prognostic prediction, a prognostic model called the PIMET score was developed. Patients were stratified into three groups based on this score: PIMET-low group (without metastasis and PIVKA-II<600 mAU/mL), PIMET-int group (with metastasis or PIVKA-II>600 mAU/mL), and PIMET-high group (with metastasis and PIVKA-II>600 mAU/mL). In both the training cohort and validation cohort, the PIMET-high group showed superior OS than the PIMET-low group (60).

Osteopontin (OPN), a glycoprotein involved in tumor progression (61), has also shown potential as a prognosis indicator in HCC patients treated with Atez/Bev. In a prospective multicenter study, high pretreatment OPN levels were identified as independent predictors of PD (OR=5.444, *P=*0.012). Furthermore, in Child–Pugh class A group, the PFS was significantly shorter in the high OPN group than in the low OPN group (62).

These novel serum protein markers provide additional insights into predicting treatment effectiveness in HCC patients receiving ICIs. However, further research is still required to expand our understanding of this area (**Table 1**).

**Table 1:** Predictive biomarkers before ICIs treatment in HCC.

**biomarker**	**treatment**	**study design**	**N**	**outcome**	**ref**
**AFP**	Nivolumab (N = 67) pembrolizumab (N=32)	retrospectively	99	Baseline levels of AFP < 400 µg/L were associated with better response and longer median PFS.	(19)
**CRAFITY** **(AFP+CRP)**	Anti-PD-1/PD-L1	retrospectively	190	A lower CRAFITY score is associated with better survival and response in patients receiving anti-PD-1/PD-L1 treatment.	(22)
**ALBI**	ICIs	prospectively	341	Pre-treatment ALBI can independently predict OS.	(27)
**ALBI**	Ipilimumab combined with nivolumab or pembrolizumab	retrospectively	25	The ALBI score was negatively correlated with OS.	(28)
**ALBI and age**	ICIs plus radiotherapy	prospectively	38	ALBI score and age were identified as independent prognostic factors for PFS and OS.	(29)
**IL-6 and IFN-α**	Atezolizumab plus Bevacizumab	prospectively	34	IL-6 and IFN-α levels were negatively associated with survival.	(34)
**IL-6**	Atezolizumab plus bevacizumab	prospectively	165	Patients with high baseline IL-6 levels had a reduced response rate and worse survival.	(35)
**TGF-β**	Pembrolizumab	prospectively	28	Patients with baseline TGF-β levels higher than 200pg/mL have a poor treatment response and shortened PFS and OS.	(37)
**CD137**	Sintilimab plus IBI305	prospectively	50	Patients experiencing clinical benefit exhibited no elevated serum CD137 concentrations.	(38)
**PD-1+ and PD-L1+ peripheral granulocyte percentages**	Atezolizumab plus bevacizumab	prospectively	34	Patients identified with a low PD-1+ granulocyte percentage displayed a longer time to progress.	(43)
**NLR and PLR**	Nivolumab	retrospectively	103	NLR < 5 was associated with improved OS. Higher PLR is associated with poorer OS.	(47)
**NLR and PLR**	ICIs	retrospectively	362	Patients with NLR ≥ 5 had shorter OS, PFS, and ORR. Patients with a baseline PLR≥300 have shorter OS and PFS.	(48)
**NLR**	Pembrolizumab	prospectively	60	Low NLR was identified as a contributing factor to pembrolizumab response.	(49)
**ALI**	Anti-PD-1	retrospectively	*98*	High ALI was an independent prognostic factor for overall survival in both groups.	(51)
**PNI**	Anti-PD-1	retrospectively	442	The PNI score was an independent predictor for OS.	(52)
**PNI**	Anti-PD-1	retrospectively	35	The PNI decreased in the progressive disease group after treatment, and an increased PNI was associated with longer OS.	(53)
**MLR**	Anti-PD-1	retrospectively	34	A high MLR is correlated with a short time to progression in anti-PD-1-treated HCC patients.	(54)
**CII**	ICIs and lenvatinib	retrospectively	129	Patients with low CII levels had better responses and longer OS.	(55)
**SIRI**	Sorafenib and/or ICIs	retrospectively	352	SIRI was an independent prognostic factor. Patients with high SIRI showed a poor response to ICIs.	(56)
**CXCL9**	Atezolizumab plus bevacizumab	retrospectively	68	Baseline low serum CXCL9 (<333 pg/mL) levels may predict early PD and a worse prognosis.	(57)
**SLFN11**	Anti-PD-1	preclinical	-	SLFN11 knockdown indicates increased immunosuppressive macrophage infiltration. ICIs were more effective in patients with high serum SLFN11 levels	(59)
**PIVKA-II**	Lenvatinib plus ICIs	retrospectively	304	PIVKA-II were independent risk factors of OS.	(60)
**Osteopontin**	Atezolizumab plus bevacizumab	prospectively	70	Elevated pretreatment osteopontin levels are linked to unfavorable response and reduced PFS.	(62)

### 2.2 Predictive indicators during treatment

#### 2.2.1 AFP and its related indicators

Multiple studies have highlighted the predictive value of monitoring AFP levels during treatment with ICIs. In a retrospective study involving 235 patients with HCC undergoing anti-PD-1 monoclonal antibody treatment, reductions of more than 50% in AFP or PIVKA-II levels compared to baseline were associated with improved treatment response, OS, and PFS. The AAP score, combining AFP, abnormal coagulation factor II, and ALBI, demonstrated good predictive value. Patients with an AAP score of ≥2 points had significantly longer PFS and OS (63). In another study by Kim et al., changes in AFP levels compared to baseline at 6-10 weeks and 14-18 weeks after ICIs treatment could predict treatment efficacy. An AFP response, defined as a decrease of more than 20% in AFP levels, was associated with a high ORR of 90.9% (10/11) at 6-10 weeks and 93.8% (15/16) at 14-18 weeks. Conversely, AFP progression, indicated by an increase of more than 20% in AFP levels, was associated with a lower ORR of 1.4% and 0.0% during the same periods. Additionally, the degree of change in AFP levels compared to baseline levels at 6-10 weeks and 14-18 weeks independently predicted patient OS, with HRs of 0.360 and 0.315 at 6-10 weeks, and 2.525 and 3.908 at 14-18 weeks (64). Moreover, Zhu et al. also reported that in HCC patients treated with Atez/Bev, a decrease in AFP level by ≥75% after 6 weeks of treatment effectively distinguished between treatment responders from non-responders, while an increase in AFP level by ≤10% better distinguished between disease control and disease progression (65).

Teng et al. proposed the CAR classification by combining the pre-treatment CRAFITY score with the 6-week post-treatment AFP response: Class I patients exhibited the best OS and PFS, followed by Class II and III, with statistically significant differences (OS: not reached vs.11.1 months vs. 4.3 months, *P* < 0.001; PFS: 7.9 months vs. 6.6 months vs. 2.6 months, *P* = 0.001). Class I patients also achieved a higher ORR of 35.0% compared to 18.2% and 0.0% in Class II and III, respectively (66). Additionally, a meta-analysis showed that an early AFP response was correlated with improved OS and PFS, higher ORR, and DCR compared to non-responders (21).

In conclusion, early changes in AFP levels during treatment hold significant predictive value, and combining AFP with other biomarkers shows promise in further enhancing predictive accuracy.

#### 2.2.2 The monocyte index

Jeon et al. conducted a prospective study on HCC patients treated with nivolumab and found that the ratio of peripheral blood circulating classical monocytes on day 7 to day 0 (cMonocyte D7/D0) was significantly higher in patients with durable clinical benefit (DCB) than in those without DCB (non-DCB). Conversely, the ratio of PD-L1+ circulating classical monocytes on day 7 to day 0 (cMonocyte-PDL1 D7/D0) was significantly higher in non-DCB patients than in DCB patients. To further evaluate this relationship, a monocyte index was constructed by dividing cMonocyte D7/D0 by cMonocyte-PDL1 D7/D0. A higher index was associated with worse survival rates, serving as an independent risk factor for PFS (HR=0.37, *P=*0.01) and OS (HR=0.32, *P=*0.03) *(* 67). Additionally, in a single-arm phase 2 clinical trial of patients with HCC who failed sorafenib plus pembrolizumab treatment, the increased number of peripheral blood CD14+/CD16+ monocytes was associated with disease progression (*P=*0.002) (49). In conclusion, these results suggest that early changes in monocytes may have some significant predictive value in determining the outcomes of ICIs treatment.

#### 2.2.3 Δ-IgG

In a retrospective study involving 72 patients, Balcar et al. investigated the role of immunoglobulin levels in ICIs treatment. Among all the immunoglobulins examined, the relative change in IgG (Δ-IgG) was found to be an independent predictor of OS in multivariable analysis. Patients could be stratified into high (Δ-IgG≥+14%) vs. low (Δ-IgG<+14%) risk groups, where the high-risk group showed a median OS decrease compared to the low-risk group (6.4 vs. 15.9 months; *P* = 0.001) (68). These findings suggest that Δ-IgG levels can serve as a prognostic indicator in ICIs treatment.

#### 2.2.4 ALT, AST, and proteinuria

In a real-world study involving 268 HCC patients treated with Atez/Bev, several factors were examined for their impact on ICIs treatment. The study found that increased bilirubin levels were associated with a significantly shorter OS and PFS. The hazard ratios for OS and PFS were 2.61 (*P*=0.042) and 2.85 (*P*=0.005), respectively, in patients with increased bilirubin levels. Additionally, elevated levels of aspartate aminotransferase (AST) or alanine aminotransferase (ALT) were also associated with a significantly shorter OS and PFS. Furthermore, the study found that patients with proteinuria had a significantly longer OS, with an HR of 0.46 (95% CI: 0.23–0.92, *P* = 0.027). However, the impact of proteinuria on PFS was not specifically mentioned in the provided information (69). These findings suggest that increased bilirubin levels, elevated AST or ALT levels, and the presence of proteinuria can serve as prognostic indicators in HCC patients receiving Atez/Bev treatment. Monitoring and managing these factors may help in assessing patient outcomes and optimizing treatment strategies (**Table 2**).

**Table 2:** Predictive biomarkers during ICIs treatment in HCC.

**biomarker**	**treatment**	**study design**	**N**	**outcomes**	**ref**
**AFP**	Anti-PD-1	retrospectively	235	Patients with AFP reduction > 50% and PIVKA-II reduction > 50% had better PFS and OS.	(63)
**AFP**	ICIs	retrospectively	108	A decrease of more than 20% in AFP levels after treatment was associated with high ORR and OS.	(64)
**AFP**	Atezolizumab plus bevacizumab	prospectively	104	The degree of change in AFP levels can predict treatment efficiency.	(65)
**CAR (CRAFITY + AFP decline after 6 weeks of treatment)**	Atezolizumab plus bevacizumab	retrospectively	89	Low CAR classification which combines CRAFITY score and AFP response at 6 weeks was associated with better response and survival.	(66)
**Monocyte index**	Nivolumab	prospectively	48	Monocyte index can predict the response and survival.	(67)
**CD14+/CD16+ monocyte**	Pembrolizumab	prospectively	60	An increase in the count of peripheral blood CD14+/CD16+ monocytes was observed in patients with disease progression.	(49)
**Δ-IgG**	ICIs	retrospectively	72	Patients exhibiting a high Δ-IgG (Δ-IgG≥+14%) demonstrated a worsened overall survival.	(68)
**AST, ALT, proteinuria**	Atezolizumab plus bevacizumab	retrospectively	268	Elevated AST or ALT levels were associated with adverse effects on both PFS and OS. Proteinuria exhibited a beneficial effect on OS.	(69)

## 3 Tumor-related biomarkers

### 3.1 PD-L1

PD-L1 has been studied as a potential biomarker of response to PD-1 therapy in various types of cancer (70, 71). However, its predictive value for HCC remains controversial. In the KEYNOTE-224 study evaluating pembrolizumab in HCC, no correlation was found between ORR and PD-L1 expression on tumor cells. Instead, a relationship was observed between the response and the combined positive score (CPS) of PD-L1 expression (10). Similarly, the CheckMate040 study did not find a clear association between tumor PD-L1 expression levels and treatment response (72). However, a different study reported a link between tumor PD-L1 and plasma PD-L1/PD-1 levels and plasma interferon-gamma (IFN-γ) or interleukin-10 (IL-10) (37).

A recent meta-analysis suggested that higher PD-L1 expression levels on tumor cells and tumor proportion score were associated with a higher ORR in HCC patients treated with ICIs (73). Another meta-analysis confirmed improved ORR in PD-L1-positive patients compared to PD-L1-negative patients (26% vs. 18%, OR=1.86). However, for DCR, there was no significant difference between PD-L1-positive and PD-L1-negative patients (74).

Moreover, Mocan et al. found that high plasma PD-L1 levels were associated with shorter OS, indicating a poor prognostic biomarker for HCC. However, this study did not explore the efficacy of ICIs treatment (75). Additionally, some studies observed that HCC patients treated with sorafenib had higher PD-L1 expression levels compared with those who did not, suggesting that sorafenib treatment may influence PD-L1 expression levels (76).

Overall, due to the complex immune microenvironment of HCC, the predictive value of PD-L1 in ICIs treatment is limited. While some studies have indicated an association between PD-L1 expression and treatment response, others have found conflicting results. Further research is warranted to better understand the role of PD-L1 in estimating the response to ICIs therapy in HCC and its potential clinical implications.

### 3.2 Tumor mutational burden

Tumor mutational burden (TMB) refers to the total number of errors in somatic genetic coding, base substitutions, gene insertions, or deletions per million bases (77). TMB is believed to reflect the tumor’s ability to produce neoantigens and is related to the efficacy of ICIs treatment (78) in certain cancers such as melanoma and lung cancer (79–81). However, in the case of HCC, studies have shown inconsistent results. One study analyzed 358 HCC patients enrolled in the GO30140 phase 1b or IMbrave150 phase 3 trial and found that although the high TMB group exhibited a higher ORR compared with the medium or low TMB groups (56% vs. 35% or 17%), the difference in PFS did not reach statistical significance (82). Other studies have reported that the TMB level of liver cancer is generally low and is not associated with treatment response or PFS (83, 84). Furthermore, differences in tissue acquisition methods and regional variations inevitably limit the clinical applicability of TMB as a predictive biomarker (85), which requires further exploration of how TMB can be used to evaluate the therapeutic effect of ICIs in HCC.

### 3.3 Circulating tumor cells

Circulating tumor cells (CTCs) are cancer cells that have detached from primary tumors or metastatic sites and entered the bloodstream (86). They hold significant potential in predicting the efficacy of ICIs when measured and analyzed through liquid biopsies (87). Several studies have investigated the association between PD-L1 expression in CTCs and treatment outcomes in HCC patients.

In a prospective study of HCC patients receiving anti-PD-1 treatment, all responders demonstrated PD-L1+ CTCs at baseline, whereas only one non-responder showed PD-L1+ CTCs (88). In another phase 1 clinical trial involving advanced gastrointestinal tumors, including HCC, patients treated with anti-PD-1 antibodies were categorized into four groups based on the baseline PD-L1 expression level in CTCs: negative, low, medium, and high. Patients with high PD-L1 expression in CTCs had a significantly higher DCR than others, and DCR in patients with expression levels≥20%, the DCR is even further elevated (89). However, discordant results were observed in another study of HCC patients receiving a PD-1 inhibitor in combination with antiangiogenic therapy and radiotherapy. In this study, patients with low PD-L1+CTCs at baseline had a higher ORR (56.5% vs. 16.7%, *P*=0.007) and longer OS (not reached vs. 10.8 months, *P*=0.001) in comparison to those with high PD-L1+CTCs. Furthermore, individuals with a dynamic decrease in PD-L1+ CTC count 1 month after treatment were more likely to achieve an objective response(OR) (90).

In summary, CTCs hold promise as an underlying biomarker for highlighting the efficacy of PD-1 in the treatment of HCC. However, further research and larger-scale prospective studies are required to establish their clinical utility and consistency.

### 3.4 Circulating tumor DNA

Circulating tumor DNA (ctDNA) is defined as tumor-derived DNA fragments released into the bloodstream from apoptotic or necrotic tumor cells. It serves as a biomarker for ICIs treatment across various cancers, including HCC (91–95). In a study involving HCC patients treated with Atez/Bev, higher baseline ctDNA levels correlated with high TMB, and patients who had undetectable ctDNA after treatment had longer PFS (96). Another study showed that responders to ICIs exhibited a decrease in the mutation allele frequency of ctDNA during treatment (84).

Contrastingly, a prospective study of 85 HCC patients treated with Atez/Bev revealed that individuals with high levels of cell-free DNA (cfDNA) experienced significantly lower ORR, PFS, and OS compared to those with low cfDNA levels. Additionally, the presence of a TERT mutation independently predicted poor OS in multivariate analysis (20).

Analyzing specific characteristics of ctDNA fragments from HCC patients may provide prognostic insights. Notably, the activation of the Wnt/β-catenin pathway was associated with poorer disease control and PFS in HCC patients treated with anti-PD-1 antibodies (97). Extensive research on DNA methylation in HCC initiation and progression revealed hypermethylated DNA at specific genes (98). Shen et al. systematically screened 14 DNA methylation-driven survival-related genes, developing a risk model involving five methylation-driven genes. Specifically, DNA methylation levels of CYBYR, CYP2C9, and LAMB1 correlated significantly with overall survival in HCC patients (99). Recent studies identified PD-L1 K162 methylation as a regulator of PD1/PD-L1 interaction, influencing T cell activity suppression. Hypermethylation of PD-L1 was associated with resistance to anti-PD-1 treatment, highlighting its potential as a predictive biomarker for assessing anti-PD-1/PD-L1 therapy response (100).

Despite ctDNA’s easy obtainability and minimal invasiveness, current evidence regarding its efficacy in predicting ICIs treatment response in HCC remains insufficient. Further large-scale studies are crucial to validate its clinical utility. As technology advances and standard operating procedures are established, ctDNA is poised to become a valuable biomarker for evaluating ICIs treatment efficacy in the future (91).

### 3.5 Clinical indicators of tumor

Clinical indicators can provide valuable information about the tumor and are generally associated with the tumor’s malignant features and prognosis (101).

The presence of metastasis, which indicates that the tumor has spread to other parts of the body, has been identified as an independent risk factor for inferior OS in patients treated with lenvatinib monotherapy or lenvatinib plus ICI (60). Additionally, the presence of extrahepatic spread (metastasis outside the liver) has been associated with an inferior ORR in ICIs-treated HCC patients (102). Macrovascular invasion, which refers to tumor thrombosis in the portal vein or hepatic vein, has also been associated with poorer PFS and OS (102). In a retrospective study involving 604 HCC patients treated with ICIs after progression, intrahepatic growth, and new vascular invasion were associated with a poorer prognosis (103).

In a retrospective study aimed at constructing the nomogram for tumor response prediction, several factors were identified as independent predictors of treatment response: solitary tumor, neutropenia, and hypertension independently predicted ORR; tumor sizes less than 5 cm, a solitary tumor, prognostic nutritional indices greater than or equal to 54.3, neutropenia and fatigue were found to independently predict disease control (104).

Overall, considering clinical indicators, such as metastasis, macrovascular invasion, and other specific tumor characteristics, can provide valuable insights into predicting the outcomes of ICIs treatment in HCC patients. However, further research and validation studies are needed to establish their clinical significance and utility in guiding treatment decisions.

### 3.6 Tumor infiltrating lymphocytes and tumor-associated macrophages

Tumor infiltrating lymphocytes (TILs), lymphocytes present within and around tumor cells, have been associated with improved outcomes in HCC immunotherapy (8, 105). In a study by Liu et al., patients in the higher frequency of CD39+/CD8+ TILs group demonstrated a favorable prognosis following anti-PD-1 therapy (106). The Checkmate 040 trial revealed a non-significant trend toward prolonged survival (*P*=0.08) in Nivolumab-treated HCC patients with elevated CD3+/CD8+ TILs (107). Conversely, tumor-associated macrophages (TAMs), macrophages present within tumor tissues or clustering in the solid tumor microenvironment (TME), have been implicated in inducing immune suppression in the HCC TME, with their frequency correlating with poor prognosis (108). Recently, Qu et al. constructed an HCC prognostic model utilizing public databases, identifying eight M2-like TAM-related genes (PDLIM3, PAM, PDLIM7, FSCN1, DPYSL2, ARID5B, LGALS3, and KLF2) (109). In an anti-PD-1 treatment study involving eight HCC patients, a tumor–immune barrier structure comprising SPP1+ macrophages and cancer-associated fibroblasts was identified in non-responsive patients (110).

Despite their crucial roles in the TME, detecting and accessing TILs and TAMs pose significant challenges in clinical applications. The emergence of sophisticated detection technologies, such as single-cell RNA sequencing, holds promise for overcoming these challenges and establishing TILs and TAMs as predictive biomarkers for ICIs in HCC (111, 112) (**Table 3**).

**Table 3:** >Tumor-related biomarkers for ICIs treatment in HCC.

**biomarker**	**treatment**	**study design**	**N**	**outcomes**	**ref**
**PD-L1**	Pembrolizumab	prospectively	104	The ORR of pembrolizumab treatment was related to the combined positive score of PD-L1 expression	(10)
**PD-L1**	Nivolumab plus ipilimumab	prospectively	148	No correlation was observed between tumor PD-L1 expression and treatment response.	(72)
**PD-L1**	Pembrolizumab	prospectively	28	PD-L1 expression levels were not related to the response to ICIs treatment.	(37)
**TMB**	Nivolumab plus ipilimumab	prospectively	358	The high TMB group exhibited a higher ORR compared to the medium or low TMB groups.	(82)
**TMB**	Nivolumab plus ipilimumab	retrospectively	90	TMB was not associated with response or PFS	(83)
**CTCs**	Anti-PD-1	prospectively	10	All responders demonstrated PD-L1+ CTCs.	(88)
**CTCs**	Anti-PD-1	prospectively	35	A phase 1 clinical trial for advanced gastrointestinal tumors, including liver cancer, showed that the DCR of patients in the high PD-L1 expression group was significantly higher than that of other patients.	(89)
**CTCs**	Anti-PD-1, antiangiogenic therapy, and radiotherapy	prospectively	47	Patients with low PD-L1+ CTCs at baseline had a higher ORR and longer OS.	(90)
**ctDNA**	Nivolumab plus ipilimumab	prospectively	48	Patients who had undetectable ctDNA after treatment had longer PFS.	(96)
**ctDNA**	Anti-PD-1	prospectively	34	The combined score including Wnt/β-catenin activation, CPS of PD-L1, and degree of CD8+ TILs in HCC is informative for predicting the response to ICI in HCC cases.	(97)
**ctDNA**	Atezolizumab plus bevacizumab	prospectively	85	Patients with high cfDNA levels showed a lower ORR and shorter PFS and OS	(20)
**ctDNA**	–	preclinical	–	Hypermethylation status of PD-L1 is associated with resistance to anti-PD-1 treatment	(100)
**Intrahepatic growth and new vascular invasion**	ICIs	retrospectively	364	Intrahepatic growth and new vascular invasion were associated with shorter post-progression survival	(103)
**Nomogram**	Immunotherapy plus targeted therapy	retrospectively	221	Solitary tumor, neutropenia, and hypertension predict OR. Solitary tumor, prognostic nutritional, neutropenia, and fatigue independently predicted DC.	(104)
**TILs**	Anti-PD-1	prospectively	56	A higher frequency of CD39+/CD8+ TILs is associated with a better prognosis after pd-1 treatment	(106)
**TAMs**	–	preclinical	–	The tumor immune barrier structure composed of SPP1+ macrophages and CAFs is associated with immunotherapy resistance	(110)

## 4 Imaging and personal features

### 4.1 Splenomegaly

Splenomegaly refers to the enlargement of the spleen and is often confirmed using radiological imaging, although the “gold-standard” definition is based on splenic weight. In a retrospective analysis involving 161 liver cancer patients treated with ICIs, Xiao et al. observed that patients with splenomegaly had significantly lower OS and PFS compared to those without splenomegaly (113). The precise mechanism by which splenomegaly affects the efficacy of immunotherapy is not yet clear. Hypotheses include its potential influence on the number or function of splenic lymphocytes or its effects through the compression of adjacent abdominal organs (113). Further research is needed to establish and verify its predictive value.

### 4.2 Skeletal muscle index

The skeletal muscle index (SMI) is the ratio of the cross-sectional area of the muscles at the level of the third lumbar vertebra (L3) to the square of the height (114). It serves as an indicator of the body’s nutritional status and is commonly used to evaluate sarcopenia, which is defined as a male SMI < 43 cm^2^/m^2^ (when BMI < 25) or < 53 cm^2^/m^2^ (when BMI ≥ 26), or female SMI < 41 cm^2^/m^2^ (115, 116). A retrospective study demonstrated that sarcopenia was significantly associated with adverse survival outcomes with anti-PD-1/PD-L1 treatment (HR=5.39, *P=*0.004). Interestingly, the study did not find a similar relationship between BMI and survival outcomes (117). Furthermore, another study conducted by Matsumoto et al. found that in patients treated with Atez/Bev, those who did not experience a decline in SMI had significantly longer PFS compared to those with a decline in SMI (8.5 months vs. 5.8 months), while baseline sarcopenia did not show a significant association with survival outcomes in this study (118). It is worth noting that a previous retrospective study observed a trend toward worse OS in patients with sarcopenia, although statistical significance was not reached (119).

In summary, changes in muscle mass, indicated by a decline in SMI, have the potential to predict the efficacy of ICIs treatment. Further research is necessary to better understand the relationship between sarcopenia and survival outcomes in HCC. Monitoring muscle changes using SMI can provide valuable information regarding the prognosis of HCC patients.

### 4.3 BMI

Obesity is generally recognized as having adverse effects on health and clinical outcomes (120). However, recent studies have suggested that obesity may be associated with an improved response to ICIs in various cancer types, such as melanoma and non-small cell lung cancer (NSCLC), and it may also impact the dosing strategy of ICIs (121–123). Nevertheless, the specific effect of obesity on immunotherapy outcomes in HCC is still unclear. One retrospective study focused on HCC patients treated with anti-PD-1 antibodies found that median OS was 5 months in patients with a BMI of less than 25, while it was 17.5 months in patients with a BMI of 25 or greater (Log-rank *P=*0.034) (119). On the other hand, another study involving 191 HCC patients treated with Atez/Bev found that overweight (BMI ≥25) patients had similar OS compared to non-overweight patients. Additionally, BMI did not significantly influence median PFS, ORR, and DCR in this study (124).

In summary, some studies suggest a potential association between obesity and an improved response to ICIs, while others did not find significant correlations. Further research is needed to better understand the relationship between obesity and ICIs treatment outcomes in HCC.

### 4.4 Age

Several studies have reported comparable efficacy of ICIs in younger and older patients across various cancer types (125–127). However, in HCC, interestingly, age has been found to be a prognostic factor in patients treated with ICIs. The Keynote-240 phase III trial, which investigated pembrolizumab in HCC, conducted a subgroup analysis of patients aged 65 years or older and revealed that immunotherapy with pembrolizumab improved PFS compared to placebo in this age group (11).

Furthermore, in HCC patients receiving ICIs combined with radiotherapy treatment, older patients aged 65 years or older showed better responses to ICIs in terms of both PFS (HR=0.955, *P=*0.037) and OS (HR=0.931, *P<*0.001) (29).In another multicenter retrospective study involving 540 patients treated with ICIs, patients aged 65 years or older responded better to ICIs in terms of DCR and PFS, while maintaining similar ORR and OS (128). The analysis of public datasets revealed that the elderly group had lower expression of oncogenic pathways such as PI3K-Akt, Wnt, and IL-17, and higher tumor mutation burden compared to younger patients (128).

The gene characteristics may explain the results to some extent; however, whether age is an independent prognostic factor remains controversial, as older age may also influence liver function (129). Further research is necessary to fully understand the underlying mechanisms and validate the predictive role of age in HCC patients treated with ICIs (**Table 4**).

**Table 4:** Imaging and host-related biomarkers.

**biomarker**	**treatment**	**study design**	**N**	**outcomes**	**ref**
**Splenomegaly**	ICIs	retrospectively	161	Splenomegaly is linked to significantly lower OS and PFS.	(113)
**SMI**	ICIs	retrospectively	172	Sarcopenia was significantly associated with adverse survival outcomes.	(117)
**SMI**	Atezolizumab plus bevacizumab	prospectively	32	No decline in SMI linked to significantly longer PFS.	(118)
**BMI**	anti-PD-1	retrospectively	57	BMI ≥25 is associated with longer OS.	(119)
**BMI**	Atezolizumab plus bevacizumab	retrospectively	191	Being overweight is not associated with patient response or prognosis.	(124)
**Age**	ICIs	retrospectively	540	Patients aged 65 years or older responded better to ICIs in terms of DCR and PFS.	(128)

## 5 Etiology

### 5.1 Non-alcoholic steatohepatitis

Studies have indicated that patients with HCC associated with non-alcoholic steatohepatitis (NASH) did not experience improved survival after treatment with ICIs. A retrospective study involving 75 HCC patients treated with ICIs demonstrated significantly higher rates of disease progression in patients with NASH cirrhosis compared to those without NASH cirrhosis (130). Additionally, Pfister et al. reported decreased survival in NASH-induced HCC after ICIs treatment compared to HCC caused by other factors (131). The underlying mechanisms include a loss of antitumor CD4+ T cells, and an accumulation of exhausted, unconventionally activated CD8+PD-1+ T cells, which impede tumor immune surveillance and the efficacy of immunotherapy (131, 132). Moreover, NASH-cirrhosis patients exhibit microbiome disorders and changes in the intestinal microbiome of NASH-associated HCC patients may contribute to peripheral immunosuppression, thereby impairing the efficacy of ICIs (133).

### 5.2 HBV and HCV

Hepatitis B virus (HBV) and Hepatitis C virus (HCV) infections significant risk factors for HCC. Phase III studies suggest that ICIs treatment may be more effective in virus-related HCC compared to non-viral-related HCC. In the IMbrave150 trial investigating Atez/Bev in HCC, the median OS of patients treated with this combination therapy was shorter in nonviral-related HCC compared to HBV- and HCV-related HCC (16). The HIMALAYA study also showed improved OS in patients with HBV who received durvalumab plus tremelimumab compared to sorafenib (HR=0.64), while this improvement was not observed in HCV-related HCC (134).

Recent meta-analyses have further supported the notion that viral infections are associated with a better prognosis after ICIs therapy in HCC. One meta-analysis of eight trials involving 3739 patients showed that ICI therapy was significantly more effective in patients with viral hepatitis compared to non-viral-related HCC (135). Another meta-analysis found that patients with viral infections achieved a better prognosis than those without infections (*P* = 0.018), particularly in HBV-associated HCC (*P* = 0.016), but not in HCV-associated HCC (*P* = 0.081) (136). However, there have been conflicting results, as another meta-analysis did not find a significant effect of viral etiology on ICIs treatment in HCC (137).

A study revealed that among HBV-related liver cancer patients receiving ICIs combined with TKIs treatment, patients with disease control (DC) showed a more significant decrease in HBV DNA and HBsAg levels compared to patients with PD. Higher levels of HBV DNA (HR=4.816, *P=*0.011) and HBsAg (HR=4.161, *P=*0.022) were identified as independent risk factors for survival, suggesting that higher levels of HBV DNA and HBsAg indicate poorer tumor response and outcomes (138). However, the study also found that regardless of baseline HBV DNA levels, the tumor response of HBV-related HCC patients was similar to that of other HCC patients. This suggests that a high viral load should not be a contraindication for ICIs treatment in HBV-related liver cancer patients and antiviral treatment may benefit these patients by reducing the risk of hepatic decompensation (139–141).

Overall, virus-related HCC, especially HBV-related HCC, may serve as a prognostic factor in ICIs treatment. However, the relationship between viral etiology and the response to ICIs in HCC is complex and requires further investigation (**Table 5**).

**Table 5:** Cause-related biomarkers.

**biomarker**	**treatment**	**study design**	**N**	**outcomes**	**ref**
**NASH**	Anti-PD-1/PD-L1	preclinical	-	Immune therapy did not improve survival in patients with NASH-related HCC.	(131)
**NASH**	ICIs	retrospectively	79	NASH patients had a higher rate of disease progression.	(130)
**HBV DNA and HBsAg levels**	Anti-PD-1 and TKIs	retrospectively	48	Elevation of HBV DNA and HBsAg levels indicate poorer tumor response and survival time.	(138)

## 6 Gut microbiome

The gut microbiota engages in profound interactions with the host’s immune system, and its polymorphic nature is considered one of the hallmarks of cancer (19, 142). Research has demonstrated the influence of gut microbiota on the effectiveness of ICIs in various cancers (143–146). As ICIs therapy continues to evolve, the emerging importance of the gut microbiome in HCC is becoming evident. In a prospective study by Lee et al., the relationship between pre-treatment fecal samples and ICIs treatment in HCC was investigated. The study found that the composition of fecal bacteria before treatment significantly correlated with the therapeutic response (147). *Prevotella 9* was enriched in PD patients, while *Lachnoclostridium*, *Lachnospiraceae*, and *Veillonella* were more dominant in OR patients. Patients with a more favorable microbiota composition, characterized by the absence of *Prevotella 9* and enrichment of *Lachnoclostridium*, had better PFS and OS. Additionally, specific metabolites, such as ursodeoxycholic acid and ursocholic acid, were enriched in the feces of patients who responded well to treatment, suggesting a potential relationship between pre-treatment fecal microbiota, bile acids, and the outcome of HCC immunotherapy (147).

Moreover, changes in gut microbiota during treatment may also predict treatment outcomes. In a prospective study involving patients receiving tremelimumab and/or durvalumab treatment, *Akkermansia* was enriched in patients who achieved disease control, while *Enterobacteriaceae* decreased (148). However, another study by Shen et al. did not find significant changes in gut microbiota between pre-ICIs treatment and 8 weeks post-treatment (149).

Antibiotic use can significantly impact gut microbiota in clinical settings. The alteration of gut microbial composition and function due to antibiotic use may reduce microbial diversity and adversely affect the immune response. A retrospective study with 105 HCC patients showed that those who did not receive antibiotics had longer median PFS (9.1 months vs. 3.0 months; *P*=0.049) and OS (not reached vs. 11.4 months; *P*=0.015) compared to the group that received antibiotics (150). Furthermore, a meta-analysis of 1056 patients showed that the use of antibiotics can modify the treatment effect of ICIs in HCC patients, suggesting that early exposure or use of antibiotics during treatment may affect gut microbiota and, consequently, treatment efficacy (151).

Nevertheless, findings from a retrospective study with 441 patients treated with Atez/Bev indicated that after adjusting for imbalances in baseline patient characteristics, the differences in PFS and OS between patients with and without antibiotic treatment were not statistically significant (median PFS, 3.8 vs. 6.7 months, *P=*0.2; 1-year survival rate, 61.8% vs. 71.0%, *P=*0.6) (152). Additionally, the study observed no statistically significant difference in PFS and OS between patients with and without proton pump inhibitors (PPI)(median PFS, 7.0 vs. 6.5 months, *P=*0.07, 1-year survival rate 66.3% and 73.8%, *P=*0.9) (152).

These conflicting results underscore the necessity for further research and large-scale studies to better understand the role of gut microbiota in predicting treatment response and to validate its potential as a therapeutic target. Future investigations may focus on elucidating the underlying mechanisms of gut microbiota in HCC and exploring strategies to modulate the microbiota to enhance treatment outcomes (153) (**Table 6**).

**Table 6:** Gut microbiome biomarkers.

**biomarker**	**treatment**	**study design**	**N**	**outcomes**	**ref**
**Intestinal microbiome**	ICIs	prospectively	41	*Prevotella 9* was enriched in patients with PD, whereas *Lachnoclostridium*, *Lachnospiraceae*, and *Veillonella* were predominant in patients with OR.	(147)
**Intestinal microbiome**	Tremelimumab and/or Durvalumab	prospectively	11	*Akkermansia* was enriched in patients who achieved disease control, while *Enterobacteriaceae* decreased	(148)
**Intestinal microbiome**	ICIs	prospectively	36	Gut microbiome was not associated with the efficacy of ICI in patients with HCC.	(149)
**Antibiotics**	Atezolizumab plus bevacizumab	retrospectively	105	Longer PFS and OS in antibiotics (-) group	(150)
**Antibiotics**	Atezolizumab plus bevacizumab	retrospectively	441	No difference in Atezolizumab + Bevacizumab outcomes based on PPI or antibiotic treatment.	(152)

## 7 Immune-related adverse events

The safety profile of ICIs is a critical consideration in their clinical use. While ICIs can improve patient prognosis, they can also lead to immune-related adverse events (irAEs), causing damage to normal tissues and organs, and potentially resulting in treatment discontinuation or abandonment (154, 155). The reasons for irAEs occurring in certain patients are not yet clear, and emerging evidence suggests that different immunopathogenic mechanisms can lead to distinct histopathological phenotypes in affected organs (156).

Identifying risk factors for irAEs in HCC patients is crucial to promptly identify those who may not tolerate treatment-related toxicities, avoid unnecessary pain, and reduce healthcare costs (69). Several markers have been investigated as potential predictors of irAEs. The modified ALBI (mALBI) score, which further divides grade 2 of the ALBI score into grades 2a and 2b, has gained attention. In a retrospective study involving patients undergoing Atez/Bev treatment, a significant difference in baseline mALBI scores (*P=*0.02) was observed between the group that discontinued medication due to irAEs and the group that continued treatment (157). However, a systematic review did not find a significant increase in adverse events in patients with impaired liver function (30). Furthermore, Hatanaka et al. revealed that patients with a CRAFITY score of 0 exhibited a low incidence of grade≥3 irAEs, suggesting its potential as a predictive marker for irAEs (24). Additionally, in a retrospective analysis of patients treated with ICIs combined with TKIs, Yu et al. demonstrated that compared with patients who did not develop irAEs, those who developed irAEs had higher levels of CRP and IL-6 and lower levels of lymphocyte subsets (excluding natural killer cell counts), all of which may serve as potential biomarkers for irAEs (158).

The pathophysiological mechanisms underlying irAEs are believed to be related to the role of immune checkpoints in maintaining immunological homeostasis (155). Interestingly, some retrospective studies have indicated that patients who experienced irAEs exhibited improved outcomes compared to those who did not (159, 160). In a study involving 65 HCC patients who received anti-PD-1 treatment, the median PFS in the irAEs group was superior to that in the non-irAEs group. Multivariate analysis demonstrated that the absence of irAEs (HR = 6.410, *P=*0.017) independently correlated with poor prognosis (159). Another study involving 198 patients undergoing ICIs treatment found that the occurrence of grade ≥3 irAEs was associated with better survival compared to grades 1 and 2 (PFS: 8.5 months vs. 3.6 months vs. 1.3 months; OS: 26.9 months vs. 14.0 months vs. 4.6 months) (160). However, some concerns have been raised by other researchers regarding the grouping and statistical analysis methods employed in this study (161). Furthermore, in a multicenter study involving 268 patients treated with Atez/Bev, no significant differences were found in OS and PFS concerning irAEs (69). These conflicting results show that further research is needed to fully understand the relationship between irAEs and treatment outcomes in HCC patients. Larger-scale studies are necessary to clarify the predictive value of irAEs in the context of ICIs treatment for HCC (**Table 7**).

**Table 7:** irAE-related biomarkers.

**biomarker**	**treatment**	**study design**	**N**	**outcomes**	**ref**
**mALBI**	Atezolizumab plus bevacizumab	retrospectively	28	Pretreatment mALBI grades 1 and 2a were predictive factors associated with the irAE and the therapeutic continuation.	(157)
**CRAFITY score**	Atezolizumab plus bevacizumab	retrospectively	297	The percentage of patients with grade ≥ 3 irAE was lowest in patients with a CRAFITY score of 0, followed by patients with CRAFITY scores of 1 and 2.	(24)
**CRP, IL-6, and lymphocyte subsets**	Anti-PD-1 and TKIs	retrospectively	67	Elevated CRP and IL-6 levels, coupled with reduced levels of lymphocyte subsets, were linked to the onset of irAEs.	(158)
**irAE**	Atezolizumab plus bevacizumab	retrospectively	268	The OS and PFS were not different in terms of adverse events.	(69)
**irAE**	ICIs	retrospectively	168	The presence of grade ≥3 irAEs was associated with a longer OS.	(160)
**irAE**	Anti-PD-1	retrospectively	65	The absence of irAEs correlated with poor prognosis.	(159)

## 8 Discussion

Despite significant advancements in HCC treatment in recent years, the overall five-year survival of patients remains unsatisfactory due to challenges in early diagnosis, treatment response prediction, and treatment response. Therefore, there is a pressing need to explore robust biomarkers that can improve the efficacy of ICIs in HCC treatment. Currently, biomarkers for predicting ICIs response in HCC are still in the exploration stage and lack compelling evidence. The existing studies on the predictive efficacy of ICIs are limited, often comprising small sample sizes and retrospective designs. To establish stronger evidence, larger prospective studies are warranted.

Although certain potential biomarkers, such as PD-L1 and TMB, have been identified, their predictive value in HCC is limited. On the other hand, easily accessible serum biomarkers like NLR, ALBI, and the CRAFITY score offer promising methods for predicting the efficacy of ICIs, and combining multiple factors may enhance the accuracy of these predictions. Novel biomarkers such as CTCs and ctDNA have the potential to precisely reflect the preexisting immunity within the tumor tissue. However, their practical application is currently hindered by the lack of standardized procedures and reliable measurement technologies (162). The gut microbiome has emerged as another promising biomarker for HCC. With the development of fecal microbiota transplantation, it may even be utilized as a combination treatment to enhance the efficacy of ICIs in the future. Nevertheless, further research is necessary to unravel the underlying mechanisms.

Artificial intelligence (AI) and machine learning (ML) represent a transformative era in HCC immunotherapy, offering superior prognostic biomarkers compared to conventional techniques. The capacity of AI and ML to analyze vast datasets, encompassing radiology, pathology, genomics, and proteomics presents an unprecedented opportunity for precision medicine and personalized treatment (163, 164). Yet, practical implementation confronts nonnegligible challenges such as data privacy, model interpretability, and rigorous clinical validation (165, 166). Striking a balance between harnessing the power of AI, safeguarding patient privacy, enhancing model transparency, and ensuring real-world applicability through thorough validation are crucial steps in realizing the full potential of AI and ML in HCC prognosis and treatment (167).

In conclusion, the response and prognosis of ICIs treatment in HCC are influenced by various factors, including intrinsic characteristics of tumor tissue, the TME, and host immunity. Future efforts should focus on exploring novel biomarkers, and the management of ICIs treatment in HCC patients should involve dynamic monitoring and personalized evaluation, taking into account multiple indicators to maximize the role of ICIs and maximize the benefits for patients.

## Author contributions

RQ: Data curation, Writing – original draft. TJ: Conceptualization, Funding acquisition, Writing – review & editing. FX: Conceptualization, Funding acquisition, Writing – review & editing, Investigation, Supervision.

## Glossary

**Table:**

ALT	Alanine aminotransferase
ALBI	Albumin-bilirubin
AFP	Alpha-fetoprotein
PIVKA-II	Vitamin K absence or Antagonist-II
AI	Artificial intelligence
AST	Aspartate aminotransferase
Atez	Atezolizumab
Bev	Bevacizumab
BMI	Body mass index
cfDNA	Cell-free DNA
CII	Circulating immune index
CTCs	Circulating tumor cells
ctDNA	Circulating tumor DNA
CPS	Combined positive score
CAR	CRAFITY score and AFP response
CRP	C-reactive protein
CTLA-4	Cytotoxic T lymphocyte-associated antigen-4
DC	Disease control
DCR	Disease control rate
DCB	Durable clinical benefit
HBV	Hepatitis B virus
HCV	Hepatitis C virus
HCC	Hepatocellular carcinoma
ICIs	Immune checkpoint inhibitors
irAEs	Immune-related adverse events
IFNα	Interferon alpha
IL-6	Interleukin 6
ML	Machine learning
MLR	Monocyte-to-lymphocyte ratio
NLR	Neutrophil to lymphocytes ratio
NASH	Non-alcoholic steatohepatitis
NSCLC	Non-small cell lung cancer
ORR	Objective response rate
OR	Objective response
OPN	Osteopontin
OS	Overall survival
PLR	Platelet lymphocyte rate
PNI	Prognostic nutritional Index
PD-1	Programmed cell death -1
PD-L1	Programmed cell death ligand-1
PFS	Progression-free survival
PD	Progressive disease
SMI	Skeletal muscle index
SIRI	Systemic inflammation response index
TGF-β	Transforming growth factor-β
TAMs	Tumor-associated macrophages
TILs	Tumor infiltrating lymphocytes
TMB	Tumor mutational burden
TME	Tumor microenvironment
TKIs	Tyrosine kinase inhibitors
VEGFR	Vascular endothelial growth factor receptor.
